# Jitter-Caused Clutter and Drift-Caused Clutter of Staring Infrared Sensor in Geostationary Orbit

**DOI:** 10.3390/s23115278

**Published:** 2023-06-02

**Authors:** Boyuan Bian, Feng Zhou, Xiaoman Li

**Affiliations:** Beijing Institute of Space Mechanics & Electricity, Beijing 100094, China

**Keywords:** geostationary orbit, staring infrared sensor, jitter-equivalent angle, jitter-caused clutter, drift-caused clutter

## Abstract

For staring infrared sensors in geostationary orbit, the clutter caused by the high-frequency jitter and low-frequency drift of the sensor line-of-sight (LOS) is the impact of background features, sensor parameters, LOS motion characteristics, and background suppression algorithms. In this paper, the spectra of LOS jitter caused by cryocoolers and momentum wheels are analyzed, and the time-related factors such as the jitter spectrum, the detector integration time, the frame period, and the temporal differencing background suppression algorithm are considered comprehensively; they are combined into a background-independent jitter-equivalent angle model. A jitter-caused clutter model in the form of multiplying the background radiation intensity gradient statistics by the jitter-equivalent angle is established. This model has good versatility and high efficiency and is suitable for the quantitative evaluation of clutter and the iterative optimization of sensor design. Based on satellite ground vibration experiments and on-orbit measured image sequences, the jitter-caused clutter and drift-caused clutter models are verified. The relative deviation between the model calculation and the actual measurement results is less than 20%.

## 1. Introduction

Staring infrared sensors in geostationary orbit can achieve the high-frame-rate detection of fixed positions and have the advantages of high spatial resolution, high temporal resolution, and high sensitivity. They play an important role in the field of infrared point target detection. Under temporal infrared point target detection, the sensor noise and the clutter caused by the line-of-sight (LOS) motion are important factors that affect the sensor detection performance. Sensor noise is basic and unavoidable, caused by various independent noise sources; due to the influence of factors such as the detector material and manufacturing process, the suppressible level of sensor noise is limited. Due to the inhomogeneity of the spatial distribution of the background radiation, the LOS motion leads to changes in the radiant energy on the detector, resulting in jitter-caused (high-frequency) and drift-caused (low-frequency) clutter. With the continuous improvement of the sensor detection sensitivity and spatial resolution requirements, the impact of LOS motion on sensor detection performance is increasing. Therefore, quantitative modeling of LOS-motion-caused clutter is useful for the prediction of system noise and the adjustment of the sensor parameters.

The clutter caused by LOS motion has been studied by many scholars. Cota [1] proposed that LOS motion can convert the spatial variation in background radiation into temporal clutter and divided the clutter-equivalent target (*CET*) into two categories: the drift-equivalent target and the jitter-equivalent target. The drift-equivalent target refers to the detector output fluctuation caused by the LOS drift; the jitter-equivalent target refers to the detector output fluctuation caused by the random shaking of the LOS. Lawrie [2,3] analyzed the relationships between the LOS motion rate, field of view (FOV), and LOS agility. Pohlman [4,5] evaluated the noise and clutter sources of spaceborne staring sensors, including detector noise, photon noise caused by background radiation, LOS instability, and the movement of clouds. Rapier [6,7,8] proposed a fast estimation and approximation method for *CET* and described *CET* as a function of the background power spectral density, optical system parameters, detection distance, and LOS motion rate. Myers [9] simulated high-reflectivity backgrounds in the short-wave and mid-wave infrared spectrum and analyzed the relationship between the *CET*, ground sample distance (*GSD*), LOS drift rate, and jitter amplitude. Fraedrich [10] evaluated the clutter suppression ability of the temporal differencing method and analyzed the relationship between the clutter suppression factor, LOS motion rate, detection distance, instantaneous field of view (IFOV), and frame period. Casey [11] showed a modeling method to provide feedback on the design through trade-off analysis, e.g., a staring infrared sensor that detects moving objects using the frame-differencing method can improve the performance by reducing jitter; however, the potential increased cost needs to be considered.

Typically, for instruments performing the precise imaging of celestial objects or remote sensing of the Earth’s surface, jitter is a critical requirement for high-quality imaging [12,13]. This is especially important for missions carrying high-performance optical sensor payloads with severe pointing stability requirements [14,15]. The influence of jitter on imaging has been studied by many scholars [16,17,18,19]. In terms of the jitter-caused clutter model, Schroeder [20] and Lee [21] analyzed the one-dimensional jitter of point source detectors in the background of power spectrum description. Liang [22] analyzed the one-dimensional jitter in the background and proposed a method of quantitatively evaluating the impact of one-dimensional LOS jitter on sensor performance (signal-to-noise ratio degradation). However, these articles did not consider the impact of two-dimensional jitter and *GSD*. Hu [23] considered these factors but only used the power spectrum model to describe the background and did not consider the spatial distribution of jitter-caused clutter. However, in the process of building the above clutter models, it is generally assumed that the jitter is in the form of single-frequency sinusoidal or Gaussian white noise, which is different from the amplitude–frequency curve of the actual LOS jitter, resulting in the limited accuracy of the clutter model. Therefore, it is necessary to measure the LOS jitter spectrum, which has practical engineering guiding significance, and analyze its influence on the detection performance.

In this paper, Section 2 analyzes the relationship between the radiant flux gradient on the detector and the background radiation intensity gradient and derives the equation that correlates the radiant flux distribution on the focal plane with the background radiation intensity distribution. The time-related factors of the sensor system affecting the jitter-caused clutter mainly include the jitter power spectral density, detector integration time, frame period, and temporal background suppression algorithm. Section 3 combines them into the jitter-equivalent angle, and derives the background-independent jitter-equivalent angle model for each detector. Section 4 expresses the average jitter-caused clutter intensity over the focal plane as a simple product of the jitter-equivalent angle and the RMS background radiation intensity gradient. Then, the angular displacement caused by LOS drift is shown to be equivalent to the jitter-equivalent angle, and the drift-caused clutter model is established. Section 5 validates the jitter-caused clutter and drift-caused clutter model through actual measurements. Section 6 discusses the influencing factors of jitter-caused clutter and puts forward optimization suggestions.

## 2. Background Radiation Intensity Gradient Statistics

### 2.1. Radiant Flux Gradient on the Detector

In this section, the jitter is described as the two-dimensional time-varying angular deviation $\left(\theta_{x}\left(t\right),\theta_{y}\left(t\right)\right)$ of the LOS from the reference position $\theta_{x}=\theta_{y}=0$. In the focal plane coordinates $\left(x,y\right)$, $\left(\theta_{x}\left(t\right),\theta_{y}\left(t\right)\right)$ corresponds to the image displacement $\left(\Delta x\left(t\right),\Delta y\left(t\right)\right)$, where $\Delta x\left(t\right)=f_{o}\theta_{x}\left(t\right)$, $\Delta y\left(t\right)=f_{o}\theta_{y}\left(t\right)$. Then, $P\left(x,y,t\right)$ is given by

(1) $$P\left(x,y,t\right)=\iint_{\begin{array}{l} Detector\\ Area \end{array}}E\left(x^{\prime },y^{\prime },t\right)dx^{\prime }dy^{\prime }.$$

$t_{0}$ is the reference time of $\Delta x\left(t_{0}\right)=\Delta y\left(t_{0}\right)=0$. When jitter translates the image with $\left(-\Delta x,-\Delta y\right)$ at time t, $E\left(x^{\prime },y^{\prime },t\right)$ is given by

(2) $$E\left(x^{\prime },y^{\prime },t\right)=E\left(x^{\prime }+\Delta x,y^{\prime }+\Delta y,t_{0}\right).$$

Therefore, the temporal variation in irradiance at a fixed point $\left(x^{\prime },y^{\prime }\right)$ on the focal plane can be related to the spatial variation in irradiance at time $t_{0}$. From Equations (1) and (2),

(3) $$\begin{array}{cc} P\left(x,y,t\right) & =\iint_{\begin{array}{c} Detector\\ Area \end{array}}E\left(x^{\prime }+\Delta x,y^{\prime }+\Delta y,t_{0}\right)dx^{\prime }dy^{\prime }\\ & =P\left(x+\Delta x,y+\Delta y,t_{0}\right) \end{array}.$$

The temporal variation in radiant flux on a single detector can be related to the spatial variation at time $t_{0}$. For a small jitter displacement, the Taylor series linear approximation on the right side of the above equation is given by

(4) $$P\left(x+\Delta x,y+\Delta y,t_{0}\right)≅P\left(x,y,t_{0}\right)+\frac{\partial P}{\partial x}\times \Delta x+\frac{\partial P}{\partial y}\times \Delta y.$$

Therefore,

(5) $$P\left(x,y,t\right)-P\left(x,y,t_{0}\right)={\left.\frac{\partial P}{\partial x}\right|}_{t=t_{0}}\times \Delta x\left(t\right)+{\left.\frac{\partial P}{\partial y}\right|}_{t=t_{0}}\times \Delta y\left(t\right).$$

or, in vector notation,

(6) $$P\left(x,y,t\right)-P\left(x,y,t_{0}\right)=\vec{\nabla }P\left(x,y,t_{0}\right)\times \vec{\Delta }r\left(t\right).$$

The radiant flux change caused by the jitter displacement in the direction of $\vec{\nabla }P$ is proportional to $\left|\vec{\nabla }P\right|$, while the jitter displacement in the direction perpendicular to the gradient will not cause an irradiance change. Therefore, for two-dimensional jittering, only the jitter component parallel to $\vec{\nabla }P$ is important. Although the jitter displacement is the same for the entire focal plane, each detector corresponds to a different background region with the specific $\vec{\nabla }P$, so the response fluctuations will be different.

### 2.2. Background Radiation Intensity Gradient

For the staring infrared sensor in geostationary orbit, the radiance on a small rectangular area $\left(\Delta x_{e},\Delta y_{e}\right)$ is integrated, and the radiation intensity $L\left(x_{e},y_{e}\right)\Delta x_{e}\Delta y_{e}$ is obtained. Then, the image formed by this small area is the rectangle of $\Delta x^{\prime }\Delta y^{\prime }$ centered on $\left(x^{\prime },y^{\prime }\right)$, with average irradiance $E\left(x^{\prime },y^{\prime }\right)$. At the reference time $t_{0}$ without jitter, the relationship between the focal plane coordinates and the Earth coordinates is given by

(7) $$x^{\prime }=\frac{f_{o}}{R}x_{e},y^{\prime }=\frac{f_{o}}{R}y_{e}.$$

Similarly, the dimensions of the resulting image area are given by

(8) $$\Delta x^{\prime }=\frac{f_{o}}{R}\Delta x_{e},\Delta y^{\prime }=\frac{f_{o}}{R}\Delta y_{e}.$$

The average irradiance of the small area is the collected power divided by the imaging area; then,

(9) $$\begin{array}{cc} E\left(x^{\prime },y^{\prime },t_{0}\right) & =\frac{A\tau }{R^{2}}L\left(x_{e},y_{e}\right)\frac{\Delta x_{e}\Delta y_{e}}{\Delta x^{\prime }\Delta y^{\prime }}\\ & =\frac{A\tau }{f_{o}^{2}}L\left(x_{e},y_{e}\right) \end{array}.$$

From Equations (1), (7), and (9), the relationship between the radiant flux on the detector and the background radiance is given by

(10) $$P\left(x,y,t_{0}\right)=\frac{A\tau }{R^{2}}\iint_{\begin{array}{l} Instantaneous\\ Field\;of\;View \end{array}}L\left(x_{e},y_{e}\right)dx_{e}dy_{e}.$$

$\left|\vec{\nabla }P\right|$ is defined as

(11) $$\left|\vec{\nabla }P\right|={\left({\left(\frac{\partial P}{\partial x}\right)}^{2}+{\left(\frac{\partial P}{\partial y}\right)}^{2}\right)}^{1/2}.$$

Therefore, it is necessary to differentiate Equation (10). The *GSD* constraints on x and y are given by

(12) $$\frac{R}{f_{o}}x-\frac{\Delta x_{c}}{2}<x_{e}<\frac{R}{f_{o}}x+\frac{\Delta x_{c}}{2}.$$

(13) $$\frac{R}{f_{o}}y-\frac{\Delta y_{c}}{2}<y_{e}<\frac{R}{f_{o}}y+\frac{\Delta y_{c}}{2}.$$

The R/F factor can be extracted from the integral limit; then,

(14) $$\frac{\partial P}{\partial x}=\frac{A\tau }{Rf_{o}}\int_{y_{c}-\frac{\Delta y_{c}}{2}}^{y_{c}+\frac{\Delta y_{c}}{2}}\left[E\left(x_{c}+\frac{\Delta x_{c}}{2},y_{e}\right)-E\left(x_{c}-\frac{\Delta x_{c}}{2},y_{e}\right)\right]dy_{e}.$$

(15) $$\frac{\partial P}{\partial y}=\frac{A\tau }{Rf_{o}}\int_{x_{c}-\frac{\Delta x_{c}}{2}}^{x_{c}+\frac{\Delta x_{c}}{2}}\left[E\left(x_{e},y_{c}+\frac{\Delta y_{c}}{2}\right)-E\left(x_{e},y_{c}-\frac{\Delta y_{c}}{2}\right)\right]dx_{e}.$$

The integral values on the right-hand side of the above two equations are completely dependent on the background and *GSD*, which can be evaluated from two-dimensional image data. The background is expressed as a two-dimensional array of pixel size ΔxΔy, and $L_{m,n}$ is the radiance of the pixel in the row and column. If the IFOV corresponds to the square of k×k pixels, $\left(i,j\right)$ is the image pixels at the corners of the k×k square. In this case, where $L_{i,j}$ is the mean radiance, the gradient component in the x direction is approximately given by

(16) $$\frac{\partial P}{\partial x}=\frac{A\tau }{Rf_{o}}\sum_{p=i}^{i+k-1}\left(L_{p,j+k}-L_{p,j}\right)\Delta y.$$

The unit of ∂P/∂x is $W/\left(sr\cdot m\right)$, which can be obtained by calculating the radiation intensity $b_{m,n}$ on each pixel from

(17) $$b_{m,n}=L_{m,n}\Delta x\Delta y.$$

∂P/∂x can be re-expressed as

(18) $$\frac{\partial P}{\partial x}=\frac{A\tau }{R}\frac{1}{\Delta x}\sum_{p=i}^{i+k-1}\left(b_{p,j+k}-b_{p,j}\right).$$

Similarly, the gradient component in the y direction is approximately given by

(19) $$\frac{\partial P}{\partial y}=\frac{A\tau }{R}\frac{1}{\Delta y}\sum_{p=j}^{j+k-1}\left(b_{i+k,p}-b_{i,p}\right).$$

Equations (18) and (19), combined with the definition of the gradient, give all the information required to find $\left|\vec{\nabla }P\right|$.

**Figure 1:**
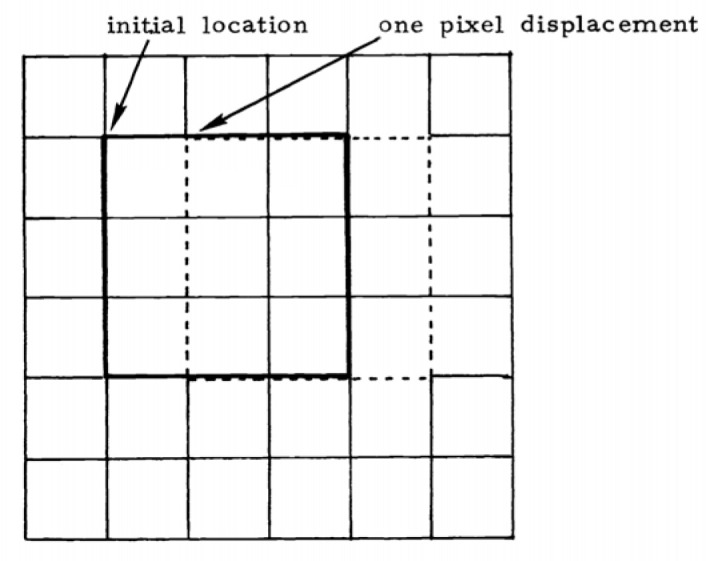
Schematic diagram before and after moving the IFOV (3 × 3) by one pixel.

The calculation result of $\left|\vec{\nabla }P\right|$ can be represented by the background radiation intensity gradient. Therefore, the background radiation intensity gradient statistic $\left|\vec{\nabla }I\right|$ is defined as the variation in background radiation intensity with unit displacement (m) within the IFOV, as shown in Figure 1. $\left|\vec{\nabla }I\right|$ is computed by

(20) $${\left|\vec{\nabla }I\right|}^{2}={\left[\frac{1}{\Delta x}\sum_{p=i}^{i+k-1}\left(b_{p,j+k}-b_{p,j}\right)\right]}^{2}+{\left[\frac{1}{\Delta y}\sum_{p=j}^{j+k-1}\left(b_{i+k,p}-b_{i,p}\right)\right]}^{2}.$$

Then, the relationship between $\left|\vec{\nabla }P\right|$ and $\left|\vec{\nabla }I\right|$ can be established. Since the jitter is described in the angular coordinates, the magnitude of the radiant flux gradient in the angular coordinates is $\left|\vec{\nabla }P\right|f_{o}$. From Equations (11) and (18)–(20), $\left|\vec{\nabla }P\right|$ can be expressed as

(21) $$\left|\vec{\nabla }P\right|f_{o}=\frac{A\tau }{R}\left|\vec{\nabla }I\right|.$$

The imaging features of the sensor are related to the background radiation intensity gradient. The equation related to the radiant flux distribution on the focal plane of the sensor to the background radiation intensity distribution has been derived in this section. The relationship between the radiant flux gradient on the detector and the background radiation intensity gradient has been analyzed.

## 3. Jitter-Equivalent Angle

### 3.1. Jitter Correlation Function

For a single detector, the jitter-caused clutter is related to the jitter spectrum, detector integration time, frame period, and background suppression algorithm. These factors can be combined into the jitter-equivalent angle $\sigma_{J}$. It is the same for different detectors.

The number of electrons $n\left(t\right)$ obtained within the integration time $T_{\mathrm{int}}$ is

(22) $$n\left(t\right)=\int_{0}^{T_{\mathrm{int}}}\frac{\lambda \eta }{hc}P\left(x,y,t^{\prime }\right)dt^{\prime }.$$

The statistical characteristics of the $n\left(t\right)$ fluctuation depend on the statistical characteristics of the jitter. $\psi \left(\tau \right)$ is the autocorrelation function of $\theta \left(t\right)$, which is called the jitter correlation function [6,7]. The autocorrelation function describes the degree of correlation at different times for a certain random signal.

Statistically, the correlation function of two temporal random variables *X*, *Y* is defined as

(23) $$corr\left(X,Y\right)=\frac{<\left(X-\mu_{X}\right)\left(Y-\mu_{Y}\right)>}{\sigma_{X}\sigma_{Y}}.$$

For a stationary random process, the autocorrelation function of the time difference *τ* is

(24) $$\psi \left(\tau \right)=\frac{<\left[X\left(t\right)-\mu \right]\left[X\left(t-\tau \right)-\mu \right]>}{\sigma^{2}}.$$

In the field of signal processing, $\psi \left(\tau \right)$ is expressed as

(25) $$\psi \left(\tau \right)=<X\left(t\right)X\left(t-\tau \right)>=\underset{T\to \infty }{\lim}\frac{1}{T}\int_{-T/2}^{+T/2}X\left(t\right)X\left(t-\tau \right)dt.$$

Therefore, from Equation (25), $\psi \left(\tau \right)$ can be defined as

(26) $$\psi \left(\tau \right)=<\theta \left(t\right)\theta \left(t-\tau \right)>.$$

From the radiation flux gradient model and Equation (6) in Section 2,

(27) $$P\left(x,y,t\right)=P\left(x,y,t_{0}\right)+\left|\nabla P\right|f_{o}\theta \left(t\right).$$

Therefore, from Equations (26) and (27), the correlation function of the radiation flux change is

(28) $$<P\left(t\right)P\left(t-\tau \right)>={\left|\nabla P\right|}^{2}f_{o}^{2}\psi \left(\tau \right).$$

### 3.2. Differenced Output of the Detector

For the background suppression algorithm of temporal differencing, background suppression refers to the differencing of image DN values or the detector outputs. If the frame period of the sensor is s, the differenced output is defined as

(29) $$n\left(s,t\right)=n\left(t\right)-n\left(t-s\right).$$

The electrical power of a signal is proportional to the square of the amplitude. The mean squared amplitude of $n\left(s,t\right)$ is [7]

(30) $$\begin{array}{cc} <n{\left(s,t\right)}^{2}> & =<{\left[n\left(t\right)-n\left(t-s\right)\right]}^{2}>\\ & =2\left[<n^{2}\left(t\right)>-<n\left(t\right)n\left(t-s\right)>\right] \end{array}.$$

Since the jitter is a stationary random process, $<n{\left(t-s\right)}^{2}>=<n{\left(t\right)}^{2}>$.

In order to calculate Equation (26), the sampling correlation function $R\left(\tau \right)$ is defined as

(31) $$R\left(\tau \right)=<n\left(t\right)n\left(t-\tau \right)>.$$

Then, the mean square differenced output is

(32) $$<n{\left(s,t\right)}^{2}>=2R\left(0\right)-2R\left(s\right).$$

From Equation (22), $R\left(0\right)$ and $R\left(s\right)$ can be expressed (see Appendix A) as

(33) $$R\left(0\right)={\left(\frac{\lambda \eta }{hc}\left|\nabla P\right|f_{o}\right)}^{2}\int_{0}^{T_{\mathrm{int}}}\left(T_{\mathrm{int}}-u\right)\left[\psi \left(u\right)+\psi \left(-u\right)\right]du.$$

(34) $$R\left(s\right)={\left(\frac{\lambda \eta }{hc}\left|\nabla P\right|f_{o}\right)}^{2}\int_{0}^{T_{\mathrm{int}}}\left(T_{\mathrm{int}}-u\right)\left[\psi \left(s+u\right)+\psi \left(s-u\right)\right]du.$$

The jitter statistics can be given by the power spectral density $\omega \left(f\right)$. The relationship between the power spectral density and the jitter correlation function is given by

(35) $$\omega \left(f\right)=4\int_{0}^{\infty }\psi \left(\tau \right)\cos2\pi f\tau d\tau .$$

(36) $$\psi \left(\tau \right)=\int_{0}^{\infty }\omega \left(f\right)\cos2\pi f\tau df.$$

Then, the sampling correlation function is expressed by the power spectral density as

(37) $$R\left(0\right)={\left(\frac{\lambda \eta }{hc}\left|\nabla P\right|f_{o}\right)}^{2}\int_{0}^{\infty }2\omega \left(f\right)\frac{1}{{\left(2\pi f\right)}^{2}}\left[1-\cos2\pi fT_{\mathrm{int}}\right]df.$$

(38) $$R\left(s\right)={\left(\frac{\lambda \eta }{hc}\left|\nabla P\right|f_{o}\right)}^{2}\int_{0}^{\infty }2\omega \left(f\right)\cos\left(2\pi fs\right)\frac{1}{{\left(2\pi f\right)}^{2}}\left[1-\cos2\pi fT_{\mathrm{int}}\right]df.$$

Finally, substituting Equations (37) and (38) into Equation (32), the mean square differenced output is

(39) $$<n{\left(s,t\right)}^{2}>={\left(\frac{\lambda \eta }{hc}\left|\nabla P\right|f_{o}\right)}^{2}T_{\mathrm{int}}^{2}\int_{0}^{\infty }\omega \left(f\right)S\left(f,s,T_{\mathrm{int}}\right)df.$$

(40) $$S\left(f,s,T_{\mathrm{int}}\right)=\frac{1}{{\left(\pi fT_{\mathrm{int}}\right)}^{2}}\left[1-\cos\left(2\pi fT_{\mathrm{int}}\right)\right]\left[1-\cos\left(2\pi fs\right)\right].$$

Therefore, the jitter-equivalent angle $\sigma_{J}$ can be expressed by the jitter power spectral density $\omega \left(f\right)$ and the transfer function *S* of the temporal differencing algorithm as

(41) $$\sigma_{J}^{2}=\int_{0}^{\infty }\omega \left(f\right)S\left(f,s,T_{\mathrm{int}}\right)df.$$

From Equation (41), since the unit of $\omega \left(f\right)$ is μrad^2^/Hz, *S* is dimensionless, and the unit of $\sigma_{J}$ is μrad. Thus, $\sigma_{J}$ is called the jitter-equivalent angle, and it describes the jitter power in the frequency passband of the integration time and background suppression algorithm.

## 4. Jitter-Caused Clutter and Drift-Caused Clutter

### 4.1. Jitter-Caused Clutter

According to Equations (21) and (39)–(41), for a single detector, the RMS differenced output is

(42) $$\sqrt{<n{\left(s,t\right)}^{2}{>}_{i,j}}=\frac{\pi D^{2}\lambda \eta \tau \sigma_{J}\left|\vec{\nabla }I\right|T_{\mathrm{int}}}{4hcR}.$$

For the entire focal plane, since all the factors in Equation (42) are the same except for $\left|\vec{\nabla }I\right|$, the RMS differenced output is

(43) $$\sqrt{<n^{2}>}=\frac{\pi D^{2}\lambda \eta \tau \sigma_{J}\sqrt{<\vec{\nabla }I^{2}>}T_{\mathrm{int}}}{4hcR}.$$

According to the relationship between radiance and the electron numbers, the average jitter-caused clutter intensity (*CET_J_*) for the entire focal plane is

(44) $$\begin{array}{rr} CET_{J} & =\frac{4hcf_{o}^{2}\sqrt{<n^{2}>}}{\pi D^{2}\lambda \eta \tau x_{d}^{2}T_{\mathrm{int}}}\Delta x_{c}\Delta y_{c}\\ & =\sigma_{J}\sqrt{<\vec{\nabla }I^{2}>}R \end{array}.$$

It can be found from Equation (44) that the jitter-caused clutter intensity is proportional to the jitter-equivalent angle $\sigma_{J}$ and the RMS background radiation intensity gradient statistic $\sqrt{<\vec{\nabla }I^{2}>}$.

Equation (44) shows how to decompose the jitter-caused clutter into several independent components. $\sigma_{J}$ contains the time-dependent characteristics of the sensor, and $\sqrt{<\vec{\nabla }I^{2}>}$ characterizes the background spatial structure. Under the same observation and system conditions, when the same sensor is used to view different backgrounds, the only variable factor in Equation (44) is $\sqrt{<\vec{\nabla }I^{2}>}$. Therefore, it is a key statistic describing the jitter-caused clutter intensity.

### 4.2. Drift-Caused Clutter

The low-frequency drift-caused clutter is directly related to the LOS drift rate v. The angular displacement caused by the LOS drift can be equivalent to the jitter-equivalent angle.

(45) $$\sigma_{D}=vT_{\mathrm{int}}.$$

The drift-caused clutter intensity (*CET_D_*) can be equivalently calculated by

(46) $$\begin{array}{cc} CET_{D} & =\sigma_{D}\sqrt{<\vec{\nabla }I^{2}>}R\\ & =vT_{\mathrm{int}}\sqrt{<\vec{\nabla }I^{2}>}R \end{array}.$$

As can be seen from Equation (46), the drift-caused clutter intensity is proportional to the LOS drift rate v, the integration time $T_{\mathrm{int}}$, and the RMS background radiation intensity gradient statistic $\sqrt{<\vec{\nabla }I^{2}>}$.

## 5. Experimental Verification

Since the jitter-caused clutter and drift-caused clutter will reduce the detection performance, predicting the clutter can contribute to the control of LOS stability. Firstly, in order to verify the clutter model, considering the diversity of influencing factors in orbit and the limitations of measurement instruments, the ground measurement experiment was developed and the LOS jitter spectrum was analyzed. Then, the drift displacement of the on-orbit image sequences was measured, and the LOS drift rate was calculated. According to the clutter model, the jitter-caused clutter and drift-caused clutter intensities of different backgrounds were calculated. Finally, with the on-orbit image sequences, the clutter intensities were counted, and the jitter-caused clutter and drift-caused clutter model were verified. The sensor parameters are shown in Table 1.

### 5.1. LOS Jitter Spectrum Analysis

The staring infrared sensor in geostationary orbit has a long integration time, and it is more susceptible to vibration than a low-orbit satellite sensor. According to the design of the satellite, the active parts mainly consist of a data transmission antenna, solar array drive mechanism (SADA), momentum wheel, and pulse tube cryocooler. The digital transmission antenna does not move during imaging. The fundamental frequency of the SADA is low, and the vibration energy is mainly concentrated in the low-frequency range of 0.2–20 Hz. According to the disturbance test of the SADA, its disturbance force is smaller than that of the cryocooler and momentum wheel. Therefore, the main vibration sources during the imaging of the staring infrared sensor in geostationary orbit are the cryocooler and the momentum wheel.

#### 5.1.1. Arrangement of Angular Displacement Sensors

**Figure 2:**
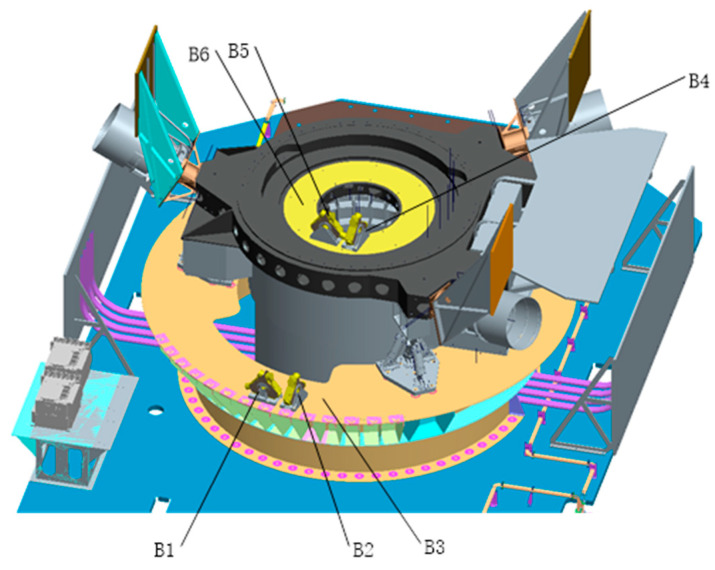
Arrangement of angular displacement sensor.

In the experiment, six angular displacement sensors are used to measure the LOS jitter characteristics. The arrangement of the measuring points is shown in Table 2 and Figure 2. The measurement frequency range of the angular displacement sensor is 1~1000 Hz. The data of the angular displacement sensors at the RX and RY positions of the primary mirror are mainly used, and the data of the integrated structure are used as an auxiliary reference.

**Figure 3:**
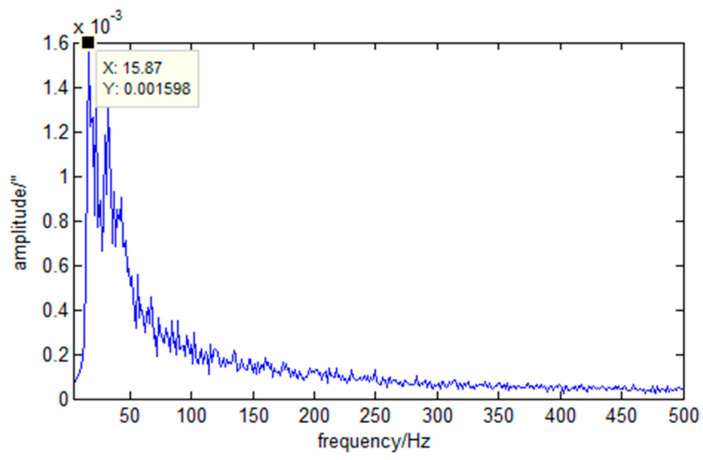
Typical noise spectrum.

The measurement principle of the angular displacement sensor is based on the Sagnac effect, and the source of error mainly includes zero value drift and random variance. Due to the vibration isolation effect of the free boundary simulation device, the high-frequency components caused by the environmental mechanical noise are attenuated and the low-frequency components are amplified. The typical noise spectrum is shown in Figure 3.

Since the disturbance frequency of the cryocooler considered in this experiment is mainly 50 Hz and its multiples, and the disturbance frequency of the momentum wheel is distributed above 20 Hz, it is necessary to reduce the noise of the angular displacement sensor. For the disturbance of the cryocooler, filtering and noise reduction were carried out through the band-selective and band-pass methods. For the disturbance of the momentum wheel, the adaptive noise cancellation method was used for noise reduction.

#### 5.1.2. Spectrum Analysis of LOS Jitter Caused by Cryocooler

The cryocooler provides low-temperature cooling for the infrared focal plane. When the cryocooler is working, the imbalance of the momentum of the moving parts such as the piston and the fluctuation in the pressure of the high-pressure gas will generate disturbance force.

**Figure 4:**
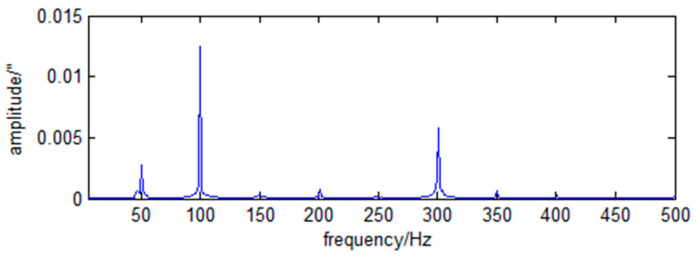
Spectrum of RX angular vibration of primary mirror caused by cryocooler.

**Figure 5:**
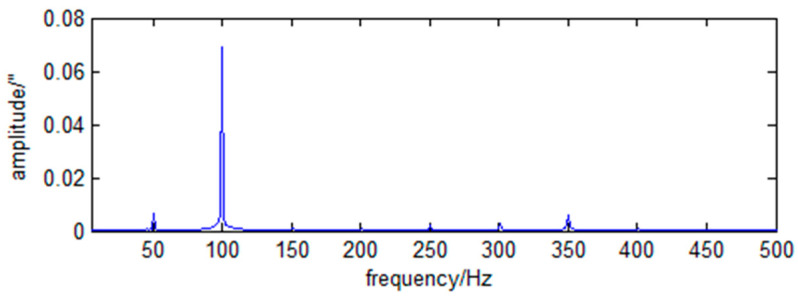
Spectrum of RY angular vibration of primary mirror caused by cryocooler.

The frequency spectra of the RX and RY direction angle vibration of the primary mirror caused by the cryocooler are shown in Figure 4 and Figure 5.

As can be seen from Figure 4 and Figure 5, the LOS jitter caused by the cryocooler is typical harmonic and has the operating frequency of the cryocooler and its multiples.

The jitter power spectral density in the RX and RY direction is approximated as

(47) $$w_{x}\left(f\right)={\left(0.015\right)}^{2}\delta \left(f-52.6\right)+{\left(0.058\right)}^{2}\delta \left(f-100.3\right)+{\left(0.03\right)}^{2}\delta \left(f-304.1\right).$$

(48) $$w_{y}\left(f\right)={\left(0.044\right)}^{2}\delta \left(f-52.6\right)+{\left(0.339\right)}^{2}\delta \left(f-100.3\right)+{\left(0.039\right)}^{2}\delta \left(f-350.9\right).$$

#### 5.1.3. Spectrum Analysis of LOS Jitter Caused by Momentum Wheels

Momentum wheels contain inertial components that rotate at high speeds to generate control torque. Due to processing errors and other reasons, the center of inertial component mass does not coincide with the actual rotation axis, resulting in unbalanced rotation and vibration. The satellite in the experiment has two momentum wheels, which are 0.5 Nm and 0.1 Nm.

**Figure 6:**
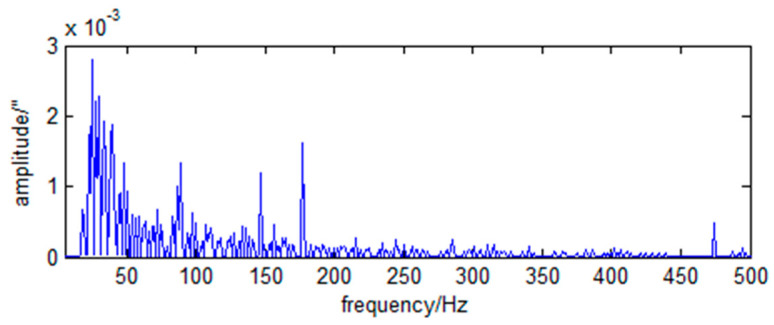
Spectrum of RX angular vibration of primary mirror caused by momentum wheels.

**Figure 7:**
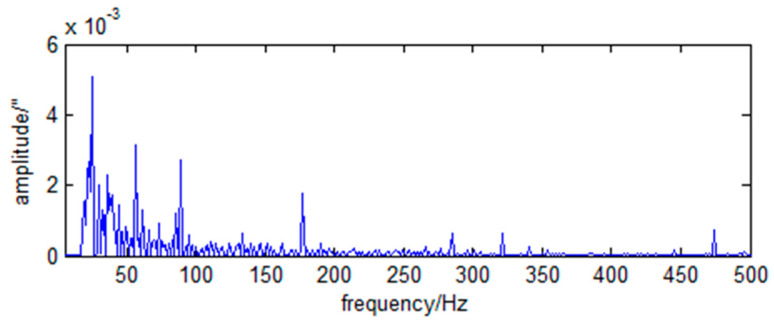
Spectrum of RY angular vibration of primary mirror caused by momentum wheels.

The frequency spectra of the RX and RY direction angle vibration of the primary mirror caused by the momentum wheels are shown in Figure 6 and Figure 7.

As can be seen from Figure 6 and Figure 7, the LOS jitter caused by the momentum wheels is typical harmonic and broadband noise.

The jitter power spectral density in the RX and RY direction is approximated as

(49) $$w_{x}\left(f\right)=\frac{0.03}{1+f^{2}}+{\left(0.006\right)}^{2}\delta \left(f-86.7\right)+{\left(0.006\right)}^{2}\delta \left(f-145.4\right)+{\left(0.008\right)}^{2}\delta \left(f-175.2\right).$$

(50) $$w_{y}\left(f\right)=\frac{0.037}{1+f^{2}}+{\left(0.015\right)}^{2}\delta \left(f-58.3\right)+{\left(0.013\right)}^{2}\delta \left(f-89.4\right)+{\left(0.008\right)}^{2}\delta \left(f-179.3\right).$$

#### 5.1.4. Comparison of Jitter-Equivalent Angle

According to the frequency spectrum of the angular vibration, the jitter-equivalent angle is calculated. The jitter-equivalent angles caused by different disturbance sources are shown in Table 3.

As can be seen from Table 3, the jitter-equivalent angle of the cryocooler disturbance is much smaller than the jitter-equivalent angle of the momentum wheel disturbance.

### 5.2. LOS Drift Rate Calculation

Based on the continuous image sequence, the LOS drift rate is approximately calculated from image geometric registration. In order to calculate the average LOS drift rate under the typical working conditions, the deviations of the on-orbit image at 10 consecutive moments are required, as shown in Table 4. Due to the limitation in the registration accuracy, it is necessary to measure the total displacement of consecutive multi-frame images and then divide it by the number of frames to obtain the displacement between two frames.

As can be seen from Table 4, between two frames of images, the average displacement of the LOS in the X direction is 0.0147 pixels. The average displacement in the Y direction is 0.0858 pixels. The RMS of the average displacements in the X and Y directions is 0.087 pixels, which is 1.45 μrad (the *IFOV* is 16.7 μrad). Therefore, the LOS drift rate is 1.45 μrad/s.

### 5.3. Results

#### 5.3.1. Typical Condition

**Figure 8:**
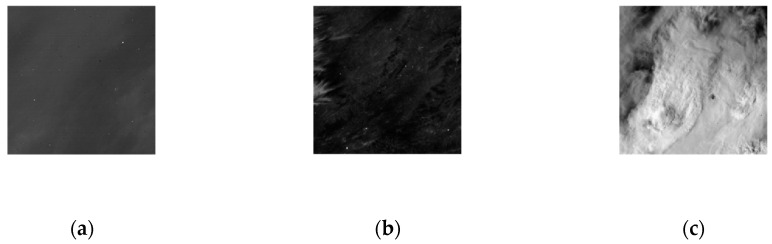
Different backgrounds. (**a**) Sea; (**b**) Land; (**c**) Clouds.

**Figure 9:**
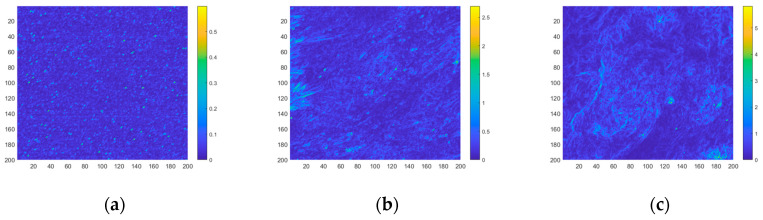
Gradient statistic distribution of different backgrounds. (**a**) Sea; (**b**) Land; (**c**) Clouds.

In this section, the clutter intensities of different backgrounds are calculated, and the jitter-caused clutter and drift-caused clutter models are verified. The sensor parameters are shown in Table 1. Three scenes are selected as typical backgrounds for measurement and analysis, as shown in Figure 8. The radiation intensity gradient statistic distribution of different backgrounds is shown in Figure 9.

#### 5.3.2. Model Calculation of Clutter Intensity

**Figure 10:**
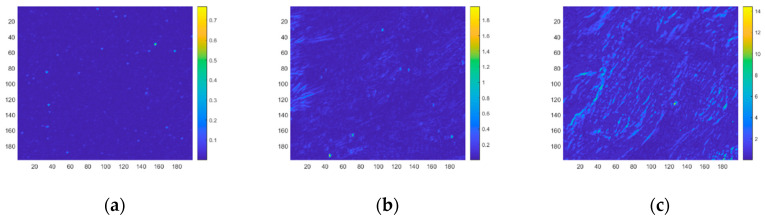
Jitter-caused clutter intensity distribution of different backgrounds. (**a**) Sea; (**b**) Land; (**c**) Clouds.

**Figure 11:**
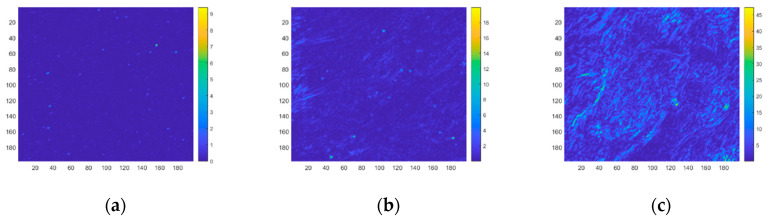
Drift-caused clutter intensity distribution of different backgrounds. (**a**) Sea; (**b**) Land; (**c**) Clouds.

According to the clutter model in Section 4, the jitter-caused clutter and drift-caused clutter intensities are calculated. The distribution of the jitter-caused clutter intensity is shown in Figure 10. The distribution of the drift-caused clutter intensity is shown in Figure 11. The jitter-caused clutter and drift-caused clutter intensities of the entire region are shown in Table 5.

As can be seen from Figure 10 and Figure 11 and Table 5, under the same system conditions, the larger the background radiation intensity gradient is, the greater the jitter-caused clutter and drift-caused clutter intensities are, which proves the rationality of the established jitter-caused clutter and drift-caused clutter models.

For the staring infrared sensor in geostationary orbit, the temporal clutter (*SET*) mainly consists of three parts: sensor temporal noise (*NET*), jitter-caused clutter (*CET_J_*), and drift-caused clutter (*CET_D_*). Therefore, SET is given by

(51) $$SET=\sqrt{NET^{2}+CET_{J}^{2}+CET_{D}^{2}}.$$

Therefore, the *SET* of three scenes calculated by the model is 11.1 W/sr, 12.4 W/sr, and 17.4 W/sr.

#### 5.3.3. Actual Measurement of Clutter Intensity

**Figure 12:**
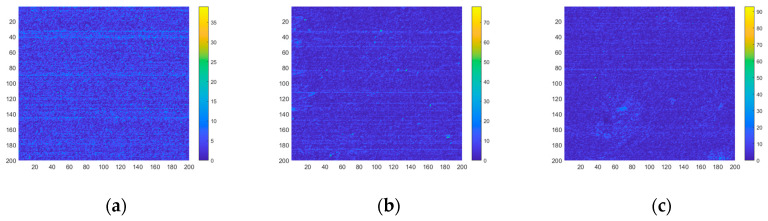
Distribution of total clutter intensity of different backgrounds. (**a**) Sea; (**b**) Land; (**c**) Clouds.

The jitter-caused clutter and drift-caused clutter models can be verified from Equation (51) and actual measurements. In addition to calculation from the model, the *SET* can also be measured from on-orbit image sequences. The distribution of *SET* in different backgrounds is shown in Figure 12, and the *SET* of the entire region is shown in Table 6.

The following can be seen from Table 5 and Table 6.

(1) The *SET* calculated by the model is similar to the actual measurement results, and the relative deviation is less than 20%. Under the same system condition, the larger the background radiation intensity gradient is, the greater the *SET* is.
(2) Under the actual LOS jitter amplitude and drift rate, for scenes with strong clutter, the background radiation intensity gradient is large. The drift-caused clutter dominates, followed by sensor noise and jitter-caused clutter. However, for scenes with weak clutter, the background radiation intensity gradient is small, and sensor noise dominates, followed by drift-caused clutter and jitter-caused clutter.
(3) The jitter-caused clutter and drift-caused clutter are related to the background radiation intensity gradient and the LOS motion. The proportion of low-frequency drift-caused clutter and high-frequency jitter-caused clutter is related to the integration time and frequency characteristics of LOS motion.

## 6. Discussion

### 6.1. Influencing Factor Analysis of Jitter-Caused Clutter

According to Equations (40), (41), and (44), the jitter-caused clutter intensity is proportional to the jitter-equivalent angle $\sigma_{J}$ and the RMS background radiation intensity gradient statistic $\sqrt{<\vec{\nabla }I^{2}>}$. The jitter-equivalent angle $\sigma_{J}$ is related to the jitter spectrum, integration time, frame period, and background suppression algorithm. The RMS background radiation intensity gradient statistic $\sqrt{<\vec{\nabla }I^{2}>}$ is related to *GSD*.

#### 6.1.1. Integration Time

**Figure 13:**
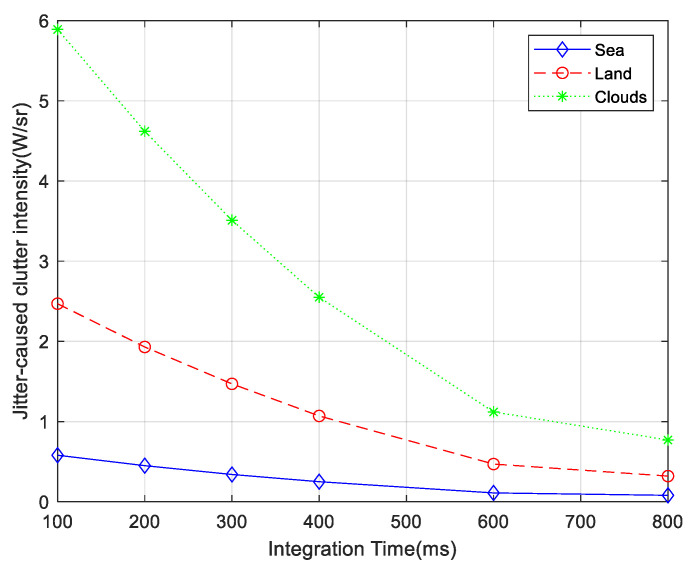
Jitter-caused clutter intensity with different integration times.

According to the jitter-caused clutter model, the jitter-caused clutter intensity of different scenes with different integration times is calculated, as shown in Figure 13.

As can be seen from Figure 13, the jitter-caused clutter intensity decreases with the increase in integration time. According to Equations (40), (41), and (44), the relationship between them is

(52) $$CET_{J}\propto \sqrt{\int_{0}^{\infty }\omega \left(f\right)\left[1-\cos\left(2\pi fs\right)\right]\frac{1-\cos\left(2\pi fT_{\mathrm{int}}\right)}{{\left(\pi fT_{\mathrm{int}}\right)}^{2}}df}.$$

#### 6.1.2. *GSD*

According to Equation (20), the background can be characterized by image pixel data. The statistic *G_k_* is defined as

(53) $$G_{k}=\sqrt{<{\left[\sum_{p=i}^{i+k-1}\left(b_{p,j+k}-b_{p,j}\right)\right]}^{2}+{\left[\sum_{p=j}^{j+k-1}\left(b_{i+k,p}-b_{i,p}\right)\right]}^{2}>}.$$

In order to compare the relative complexity of different spatial structures, the normalized RMS gradient *G_k_*/σ is used, where σ is the standard deviation of the radiant intensity of background pixels. The normalization calculation shows the spatial characteristics of the data variation, rather than the magnitude. Therefore, *GSD* is described in terms of the number of pixels instead of the dimension.

**Figure 14:**
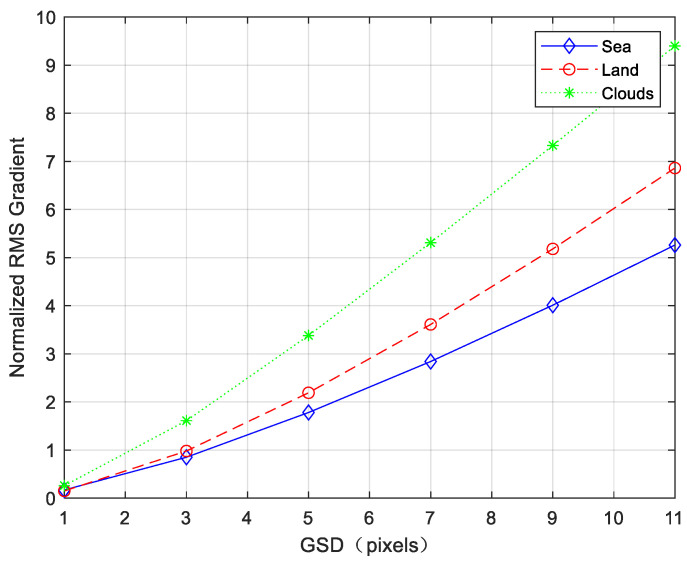
*G_k_*/σ with different *GSD*.

According to the statistical analysis of the on-orbit images, the *G_k_*/σ of different scenes with different *GSD* is calculated, as shown in Figure 14.

As can be seen from Figure 14, for different scenes, *G_k_*/σ increases with *GSD*. Therefore, from Equation (44), the jitter-caused clutter also increases with *GSD*. According to the fitting results of the power function, *G_k_*/σ is roughly proportional to the 1.5 power of *GSD*.

(54) $$G_{k}/\sigma \propto k^{1.5}.$$

The statistical and fitting results show that the power exponent depends on the spatial structure of the image. Low values occur when there are large areas with little variation in intensity, such as the sea scene, whereas high values occur for images dominated by small-scale structures, such as cloud scenes.

In order to minimize the jitter-caused clutter, the allowable jitter must be minimized and the *GSD* reduced. Further reductions in the *CET_J_* can be achieved by increasing the complexity of the background suppression algorithm.

### 6.2. Optimization Suggestions

In terms of sensor design, the requirements of the target detection performance and signal-to-clutter ratio (*SCR*) on the image are broken down into requirements of sensor noise and background clutter intensity.

The minimum target radiation intensity detectable by the system can be calculated from the threshold of *SCR* (*TCR*).

(55) $$I_{s}=TCR\times SET.$$

For staring infrared sensors in geostationary orbit with fixed parameters, *NET* is fixed. When the temporal target detection method is adopted, the LOS motion converts the spatial variation in background radiation into temporal clutter. Therefore, the LOS motion-caused clutter (*CET*) consists of two parts.

(56) $$CET=\sqrt{CET_{J}^{2}+CET_{D}^{2}}.$$

When the background, sensor parameters, and characteristics of LOS motion remain unchanged, the target detection performance can be quickly calculated, and the main factors can be judged by comparing the values of *NET* and *CET*.

NET is an important parameter to evaluate sensor performance. A smaller *NET* means better sensor performance. The sensor performance can be improved by reducing the readout noise, increasing the aperture, extending the integration time, and increasing the transmittance and quantum efficiency.

**Figure 15:**
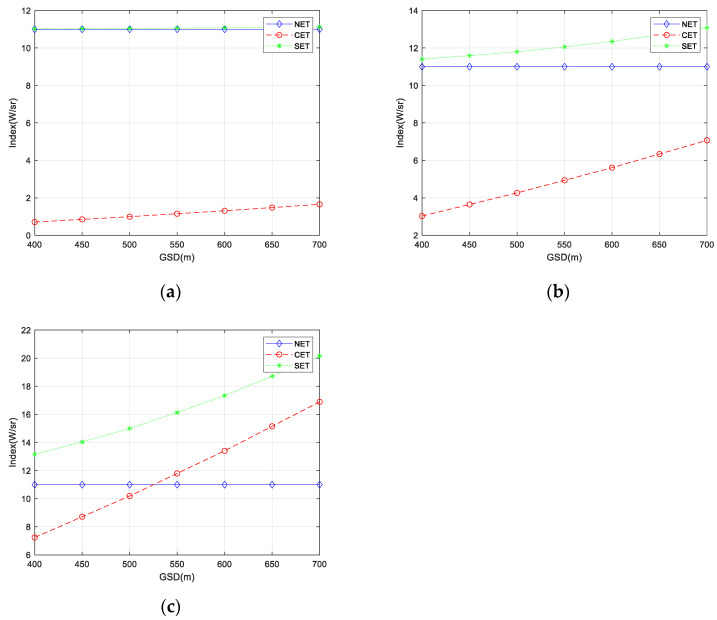
*NET*, *CET*, and *SET* with different *GSD*. (**a**) Sea; (**b**) Land; (**c**) Clouds.

According to the on-orbit image, the *NET*, *CET*, and *SET* of different backgrounds with different *GSD* are calculated, as shown in Figure 15.

As can be seen from Figure 15, for the three scenes studied in this section, as the *GSD* increases, the *CET* and *SET* increase, resulting in a decrease in the detection performance. Under different scenes, the main factors affecting the detection performance are different. For the backgrounds of sea and land, the main factor is *NET*. For the background of clouds, the main factor is different with *GSD*. When the *GSD* is less than 525 m, *NET* is the main factor; when the *GSD* is greater than 525 m, *CET* is the main factor. At this time, it is the background that mainly affects the detection performance. Improving the *GSD* can reduce the *SET* and effectively enhance the detection performance of the system. At the same time, background suppression and spectrum optimization are also primary considerations in improving the detection performance.

## 7. Conclusions

In this paper, the jitter-caused clutter of a single detector on the focal plane is analyzed, and the clutter model caused by the two-dimensional LOS jitter is established. The model shows that the jitter-caused clutter intensity of the detector is proportional to its corresponding background radiation intensity gradient, and the average jitter-caused clutter intensity of each detector is proportional to the RMS background radiation intensity gradient. Therefore, the distribution of jitter-caused clutter is related to the gradient distribution of background radiation intensity. Based on the equivalence of the LOS drift angular displacement and jitter-equivalent angle, the LOS drift-caused clutter model is established. Taking the on-orbit measured image as the background, combined with the data of the satellite ground vibration experiment and the on-orbit image displacement, the LOS jitter spectrum and LOS drift rate were analyzed, and the jitter-caused and drift -caused clutter model were verified experimentally.

The jitter-caused and drift-caused clutter model proposed in this paper provide a reference for the quantitative prediction of clutter and the control of sensor LOS stability.

## Figures and Tables

**Table 1:** The sensor parameters.

Item	Unit	Value
Average wavelength	μm	2.85
Quantum efficiency	-	0.6
Planck constant	J·s	6.626 × 10^−34^
Speed of light	m/s	3 × 10^8^
System transmittance	-	0.6
GSD	m	600
Detector size	μm	15 × 15
Optical aperture	m	0.35
Distance from sensor to Earth	km	35,786
Integration time	s	0.4
Frame period	s	1

**Table 2:** Arrangement of angular displacement measuring points.

Sensor Type	Serial Number	Location
Angular displacement sensor	B1	Primary mirror RX
	B2	Primary mirror RY
	B3	Primary mirror RZ
	B4	Integrated structure RX
	B5	Integrated structure RY
	B6	Integrated structure RZ

**Table 3:** The jitter-equivalent angles caused by different disturbance sources.

	Unit	Jitter-Equivalent Angle in the RX Direction	Jitter-Equivalent Angle in the RY Direction	Total Jitter-Equivalent Angle
Cryocooler	μrad	0.00027	0.0016	0.00163
Momentum Wheel	μrad	0.0747	0.0829	0.112

**Table 4:** LOS displacement (unit: pixels).

Direction	1	2	3	4	5
X	0.004	0.017	0.011	0.016	0.015
Y	−0.087	−0.081	−0.082	−0.071	−0.080
	**6**	**7**	**8**	**9**	**10**
X	0.018	0.012	0.008	0.030	0.016
Y	−0.110	−0.078	−0.083	−0.119	−0.067

**Table 5:** Clutter intensity of different backgrounds.

Background	RMS Background Radiation Intensity Gradient Statistics (W/sr/m)	*CET_J_* Calculated from the Model (W/sr)	*CET_D_* Calculated from the Model (W/sr)
Sea	0.0624	0.3	1.3
Land	0.2671	1.1	5.6
Clouds	0.6382	2.6	13.3

**Table 6:** Comparison of clutter intensity.

Background	RMS Background Radiation Intensity Gradient Statistics (W/sr/m)	*SET* Calculated from the Model (W/sr)	*SET* Measured (W/sr)	Relative Deviation
Sea	0.0624	11.1	13.1	15.3%
Land	0.2671	12.4	14.0	11.4%
Clouds	0.6382	17.4	16.6	4.8%

**Figure A1:**
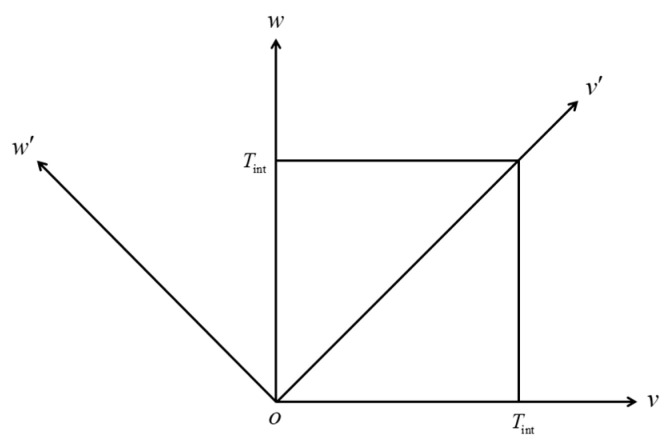
Integration area.

## Data Availability

Not applicable.

## Nomenclature

$f_{o}$ Optics’ focal length	$E\left(x^{\prime },y^{\prime },t\right)$ Irradiance at $\left(x^{\prime },y^{\prime }\right)$ on the focal plane at time *t*
$P\left(x,y,t\right)$ Radiant flux at $\left(x,y\right)$ on a single detector at time *t*	$\vec{\nabla }P$ Radiant flux gradient
$\left|\vec{\nabla }P\right|$ Gradient amplitude	$L\left(x_{e},y_{e}\right)$ Background radiance
$\left(x_{e},y_{e}\right)$ Ground coordinates	*A* Aperture area
b Background radiation intensity	τ System transmittance
$\left(x_{c},y_{c}\right)$ Center of IFOV	$\Delta x_{c}\Delta y_{c}$ *GSD* in the ground coordinate
*R* Distance from sensor to Earth	λ Wavelength
η Quantum efficiency	*h* Planck’s constant
*c* Speed of light	*D* Aperture size

## Appendix A

From the change in variables $t^{\prime }=t-T_{\mathrm{int}}+v$, Equation (22) can be expressed as

(A1) $$n\left(t\right)=\int_{0}^{T_{\mathrm{int}}}\frac{\lambda \eta }{hc}P\left(t-T_{\mathrm{int}}+v\right)dv.$$

From Equation (31), $R\left(\tau \right)$ is given by

(A2) $$R\left(\tau \right)={\left(\frac{\lambda \eta }{hc}\right)}^{2}\int_{0}^{T_{\mathrm{int}}}\int_{0}^{T_{\mathrm{int}}}<P\left(t-T_{\mathrm{int}}+v\right)P\left(t-T_{\mathrm{int}}+w-\tau \right)>dvdw.$$

From Equation (28), Equation (A2) can be expressed by $\psi \left(\tau \right)$.

(A3) $$\begin{array}{cc} R\left(\tau \right) & ={\left(\frac{\lambda \eta }{hc}\right)}^{2}\int_{0}^{T_{\mathrm{int}}}\int_{0}^{T_{\mathrm{int}}}\begin{array}{l} <P\left[\left(t-T_{\mathrm{int}}+v\right)-\left(t-T_{\mathrm{int}}+w-\tau \right)\right]\\ P\left[\left(t-T_{\mathrm{int}}+v\right)-\left(t-T_{\mathrm{int}}+w-\tau \right)-\tau \right]> \end{array}dvdw\\ & ={\left(\frac{\lambda \eta }{hc}\right)}^{2}\int_{0}^{T_{\mathrm{int}}}\int_{0}^{T_{\mathrm{int}}}<P\left(v-w+\tau \right)P\left(v-w\right)>dvdw\\ & ={\left(\frac{\lambda \eta }{hc}\left|\nabla P\right|f_{o}\right)}^{2}\int_{0}^{T_{\mathrm{int}}}\int_{0}^{T_{\mathrm{int}}}\psi \left(v-w+\tau \right)dvdw \end{array}.$$

The integration area is a square, as shown in Figure A1. The square is divided into two triangles.

The coordinates $\left(v^{\prime },w^{\prime }\right)$ are obtained by rotating coordinates $\left(v,w\right)$.

(A4) $$\left(\begin{array}{l} v^{\prime }\\ w^{\prime } \end{array}\right)=\left[\begin{array}{l} \frac{1}{\sqrt{2}}\frac{1}{\sqrt{2}}\\ -\frac{1}{\sqrt{2}}\frac{1}{\sqrt{2}} \end{array}\right]\left(\begin{array}{l} v\\ w \end{array}\right).$$

Considering the integral over the left triangle, it is not a function of $v^{\prime }$. Therefore, for a given $w^{\prime }$, the integral over the $v^{\prime }$ coordinate is the length of line segment $\sqrt{2}T_{\mathrm{int}}-2w^{\prime }$ multiplied by $\psi \left(\tau -\sqrt{2}w^{\prime }\right)$. Therefore, the integral over the left triangle is

(A5) $$R_{left}\left(\tau \right)={\left(\frac{\lambda \eta }{hc}\left|\nabla P\right|f_{o}\right)}^{2}\int_{0}^{T_{\mathrm{int}}/\sqrt{2}}\left(\sqrt{2}T_{\mathrm{int}}-2w^{\prime }\right)\psi \left(\tau -\sqrt{2}w^{\prime }\right)dw^{\prime }.$$

From the change in variables $u=\sqrt{2}w^{\prime }$,

(A6) $$R_{left}\left(\tau \right)={\left(\frac{\lambda \eta }{hc}\left|\nabla P\right|f_{o}\right)}^{2}\int_{0}^{T_{\mathrm{int}}}\left(T_{\mathrm{int}}-u\right)\psi \left(\tau -u\right)du.$$

Similarly, the integral over the right triangle is

(A7) $$R_{right}\left(\tau \right)={\left(\frac{\lambda \eta }{hc}\left|\nabla P\right|f_{o}\right)}^{2}\int_{0}^{T_{\mathrm{int}}}\left(T_{\mathrm{int}}-u\right)\psi \left(\tau +u\right)du.$$

Then,

(A8) $$\begin{array}{cc} R\left(\tau \right) & =R_{left}\left(\tau \right)+R_{right}\left(\tau \right)\\ & ={\left(\frac{\lambda \eta }{hc}\left|\nabla P\right|f_{o}\right)}^{2}\int_{0}^{T_{\mathrm{int}}}\left(T_{\mathrm{int}}-u\right)\left[\psi \left(\tau +u\right)+\psi \left(\tau -u\right)\right]du \end{array}.$$
